# Prevalence of Buruli Ulcer in Akonolinga Health District, Cameroon: Results of a Cross Sectional Survey

**DOI:** 10.1371/journal.pntd.0000466

**Published:** 2009-06-23

**Authors:** Klaudia Porten, Karen Sailor, Eric Comte, Adelaide Njikap, Agnes Sobry, Francois Sihom, Abanda Meva'a, Sarah Eyangoh, Mark Myatt, Fabienne Nackers, Rebecca F. Grais

**Affiliations:** 1 Epicentre, Paris, France; 2 Médecins Sans Frontières, Geneva, Switzerland; 3 District de Santé Akonolinga, Akonolinga, Cameroon; 4 Centre Pasteur, Yaoundé, Cameroon; 5 Division of Epidemiology, Institute of Ophthalmology, University College London, London, United Kingdom; University of Tennessee, United States of America

## Abstract

**Background:**

Buruli ulcer (BU) is a chronic, indolent necrotizing disease of the skin and underlying tissues caused by *Mycobacterium ulcerans*, which may result in functional incapacity. In 2002, Médecins Sans Frontières (MSF) opened a BU programme in Akonolinga Hospital, Cameroon, offering antibiotic treatment, surgery and general medical care. Six hundred patients have been treated in the project to date. However, due to the nature of the disease and its stigmatization, determining the exact prevalence and burden of disease is difficult and current estimates may not reflect the magnitude of the problem. The objectives of this survey were to estimate the prevalence of BU in the health district of Akonolinga, describe the geographic extension of the highly endemic area within the health district, and determine the programme coverage and its geographical distribution.

**Methodology/Principal Findings:**

We conducted a cross-sectional population survey using centric systematic area sampling (CSAS). A 15×15 km grid (quadrats of 225 km^2^) was overlaid on a map of Akonolinga district with its position chosen to maximize the area covered by the survey. Quadrats were selected if more than 50% of the quadrat was inside of the health district. The chiefdom located closest to the centre of each quadrat was selected and Buruli cases were identified using an active case finding strategy (the sensitivity of the strategy was estimated by capture-recapture). WHO-case definitions were used for nodules, plaque, ulcer, oedema and sequelae. Out of a total population of 103,000 inhabitants, 26,679 were surveyed within the twenty quadrats. Sensitivity of the case finding strategy was estimated to be 84% (95%CI 54–97%). The overall prevalence was 0.47% (n = 105) for all cases including sequelae and 0.25% (n = 56) for active stages of the disease. Five quadrats had a high prevalence of >0.6% to 0.9%, 5 a prevalence >0.3% to 0.6% and 10 quadrats <0.3%. The quadrats with the high prevalence were situated along the rivers Nyong and Mfoumou. Overall coverage of the project was 18% (12–27%) for all cases and 16% (9–18%) for active cases, but was limited to the quadrats neighbouring Akonolinga Hospital.

**Conclusions/Significance:**

Prevalence was highest in the area neighbouring the Nyong River. Coverage was limited to the area close to the hospital and efforts have to be made to increase access to care in the high prevalence areas. Use of the CSAS method was particularly useful for project planning and to identify priority areas of intervention. An added benefit of the method is that the survey procedure incorporated an awareness campaign, providing information about the disease and treatment to the population.

## Author Summary

As long as there is no strategy to prevent Buruli ulcer, the early detection and treatment of cases remains the most promising control strategy. Buruli ulcer is most common in remote rural areas where people have little contact with health structures. Information on the number of existing cases in the population and where they go to seek treatment is important for project planning and evaluation. Health structure based surveillance systems cannot provide this information, and previous prevalence surveys did not provide information on spatial distribution and coverage. We did a survey using centric systematic area sampling in a Health District in Cameroon to estimate prevalence and project coverage. We found the method was easy to use and very useful for project planning. It identified priority areas with relatively high prevalence and low coverage and provided an estimate of the number of existing cases in the population of the health district. The active case finding component of the method used served as an awareness campaign and was an integrated part of the project, creating a network of health delegates trained on Buruli ulcer.

## Introduction

Buruli ulcer (BU) is a neglected tropical disease caused by *Mycobacterium ulcerans*, belonging to the same family of organisms causing tuberculosis and leprosy. Awareness about the public health importance of the disease was raised in 1998 by the World Health Organisation (WHO) initiative [1].

BU affects predominantly children between 5 and 15 years. The clinical lesions of BU generally start as a painless subcutaneous nodule that secondarily ulcerates, presenting characteristic undermined edges. *M. ulcerans* produces a toxin, mycolactone, which destroys the skin and the subcutaneous tissues, induces necrosis and ulcerations. Ulcers are chronic, indolent and mainly located on the legs and arms. Some patients develop osteomyelitis and joint lesions. Natural evolution of the disease may lead to spontaneous healing but in the absence of early detection and appropriate treatment, the disease can extend, disseminate and leave functional incapacity [2]. Clinical diagnosis for the ulcerative form is straightforward for trained medical staff, although more difficult for the nodules, plaque and oedematous forms [3].

Based on some observational studies, the WHO recently recommended the use of the combination of Rifampicin/Streptomycin for BU treatment [4]. However, surgery remains important for BU treatment. In the early stages of infection, surgery is curative and highly cost effective, since it requires a simple excision followed by an immediate closure. In the disease's later stages, wide excisions, including healthy tissues, are needed to stop the infection and prevent recurrence or relapse at the same site. This is followed by skin grafting and requires long hospital stays [5]. As long as the mode of transmission is not understood, and in the absence of an effective vaccine, control strategies promoting early detection and treatment have achieved the best results in limiting morbidity and costs associated with the disease [6].

Although BU has been reported in 30 countries in Africa, Asia and the Western Pacific [7], determining the exact prevalence and burden of disease is difficult and current estimates may not reflect the magnitude of the problem. These difficulties include un-diagnosed cases due to fears of stigmatization, little knowledge of the disease among both the population and health workers and the variability in clinical presentation of the disease. Further, BU occurs primarily in remote rural areas where the population may have limited access to health care and the disease is not notifiable in many countries [8]. Prevalence estimates are needed for appropriate resource allocation and to plan control strategies.

In Cameroon, BU cases have been reported in 6 provinces, Adamaoua, Central, South, South-East, East and Extreme North. A national survey identified Akonolinga as a health district of high prevalence [9]. BU endemic areas are located along the Nyong River (Ayos and Akonolinga health districts) with an estimated prevalence of 0.44% in 2001 [10]. Recently, several risk factors were identified including swamp wading, wearing shorts, lower-body clothing while farming, living near cocoa plantation or wood and using adhesive bandages when hurt [11]. In 2002, Médecins sans Frontières (MSF) opened a BU project in Akonolinga District, one of the 135 health districts of Cameroon, in collaboration with the local and national health authorities of Cameroon. The project was set up in Akonolinga Hospital, with a passive case detection strategy. To date, 600 BU patients have been treated in the MSF project, which offers antibiotic treatment, surgery and general medical care. Most patients present late to the Akonolinga Hospital, presenting mainly with ulcerative lesion (about 80%) and advanced stages of BU. A study conducted in 2004 in the district described stigmatisation of BU patients and reported that traditional healers were the first source of treatment [12].

In March 2007 we conducted a cross-sectional survey to: 1) estimate the prevalence of BU in the target population of the project; 2) to estimate the proportion of BU cases visiting the MSF project at least once (coverage); and 3) to estimate the proportion of patients visiting another service provider such as a traditional healer or peripheral health centre at least once (health seeking behaviour). We also aimed to describe the spatial distribution of the prevalence as well as that of health seeking behaviour to help target the most affected areas and to address access problems for certain communities.

## Methods

### Study Site

Akonolinga health-district is a 1-hour drive from Yaoundé in the department of Nyong and Mfoumou. The health district is at its longest distance approximately 70 km east to west, and 100 km north to south. It has a surface of approximately 4500 km2. The district hospital is in Akonolinga, situated in the geographic centre of the district. The Akonolinga health-district has a total population of 103,000 inhabitants.

### Study Design

We performed a cross-sectional survey using centric systematic area sampling (CSAS). CSAS has been used successfully in past research in malnutrition and other low prevalence diseases [13]. It is particularly well suited to situations where the disease is visible, of low prevalence and where geographic distribution of prevalence and program coverage is of interest.

A 15×15 km grid (quadrats of 225 km^2^) was overlaid on a map of Akonolinga district with its position chosen to maximize the area covered by the survey. Quadrats were selected if more than 50% of the quadrat was inside of the health district, resulting in 20 quadrats identified.

Our sampling unit was the chèferie (chiefdom), the lowest administrative unit in Cameroon. The chèferie located closest to the centre of each quadrat was selected. Chèferies may be comprised of one to several villages. If the total population of the selected chèferie was below 1000 persons, the next closest chèferie was also included to obtain our required sample size as discussed below. Population information for the selected chèferies was obtained from the chief of each chèferie and crosschecked with the Chief's Office, Department of Nyong and Mfoumou and the Department of Development that compared the figures with the 2005 census. All inhabitants of the chèferies were invited to participate in the study.

To estimate an expected prevalence of all forms of BU of 0.6% with 0.1% precision, our required sample size was18,742 inhabitants. Using the same prevalence estimate, and assuming 50% program coverage with 10% precision, our required sample size was 83 cases, corresponding to a population of 13,900 persons.

We used the WHO case-definition of BU [2], limiting the definition of an active case to nodule, plaque, oedema and ulcer (Table 1). We did not include the WHO papula stages because of the very low specificity of the clinical signs and considering that this clinical form is quite rare in West Africa. Sequelae were defined as having a history of BU and complications resulting directly from the lesion (e.g., restricted limb movement, amputation, organ loss). Disfiguring stellar scars not associated with disabilities were not considered as sequelae.

**Table 1:** Clinical case definitions of active cases.

Nodule	Firm at palpation, painless, diameter at least 1 cm, in the subcutaneous tissue adherent to the skin
Plaque	A large painless swelling of at least 2 cm, with clearly marked borders, carton-like at palpation
Oedema	A large diffuse non-pitting swelling, with clearly marked borders
Ulcer	Painless, cutaneous lesion with a necrotic centre and undermined edges

Our secondary endpoints concerned program coverage, specifically attending the BU hospital, the Ministry of Health (MOH) Health Centre or a traditional healer for BU treatment. We defined “covered” as having visited the service at least once to seek treatment for the presenting BU lesion.

### Field Procedures

BU cases were identified using a combined active case finding strategy. In a preparation phase, all selected chèferies were visited. A meeting was held in Akonolinga with the chiefs of all the selected villages, to explain the survey. Health delegates from the MOH network received two days of training in survey procedures.

In each village, the survey started with a meeting, to explain the objectives of the survey and the clinical signs of BU. Villagers were informed that, on a specified day, a medical team would come and screen every suspect case of BU at a central location. Traditional healers were contacted and informed and asked to send their patients to this central screening location. They were assured that there would be no attempt to take patients away from them. Special attention was paid to ensure that key informants (women leaders, traditional healers, village leaders) understood the objectives of the survey and the different clinical forms of BU, making every effort to use non-medical terms. During the meeting, the villagers were also informed about the second part of the active case finding strategy which consisted of house to house visits by health delegates, identifying suspect cases in the household and informing them personally about the central screening. The chief introduced the health delegates identified for this task to the community.

Health delegates had at least one week between the village meeting and the day of the central screening to perform the house to house visits. They provided information about the survey and identified suspected BU cases. They discussed the fear of stigmatisation with suspected cases, and arranged individual meetings with the medical team for suspected patients who did not want to come to the central meeting point, or arranged for transport for disabled patients. They asked for oral consent of suspected cases identified for possible inclusion in the survey. They also collected information on patients who were living in a household but at the time of the survey were admitted at Akonolinga Hospital. These patients were interviewed at the hospital.

A team comprised of one doctor/nurse experienced in BU, one medical assistant and one interviewer performed the consultations and interviews at the central screening location. The lesion was inspected, measured and categorized according to clinical criteria using the clinical case definitions. A short standardized questionnaire was administered, inquiring when the first symptoms started and where the patients were seeking care. When relevant, patients were asked why they did not go to Akonolinga Hospital. Regular field visits were made to supervise the patient interviews and questionnaire procedures.

### Statistical Analysis

For the statistical analyses, CSAS was treated as a random sample [14]. Prevalence (P) was computed as (detected cases/population)*(100/sensitivity (%) of the case finding strategy)*100. Coverage was calculated as the number of detected cases who had visited the healthcare provider/total number of detected cases. Capture/recapture was used to estimate the sensitivity of the case finding strategy. The estimated total number of patients (N) was N = {[(M+1)(C+1)]/(R+1)}−1 [15] with M representing the cases detected by central location screening, C the cases detected by the combined active case finding strategy, and R cases that were detected by both strategies. Since it is impossible to have fractions of cases, the estimated value for N was rounded up to the nearest whole number [16],[17]. Sensitivity of the combined case finding strategy was computed as S = C/N.

### Qualitative Analysis

Answers to open questions in the questionnaire about health care seeking behaviour were noted word-for-word and coded in a content analysis. Resulting codes were grouped in categories.

### Ethical Considerations

Authorization to conduct the survey was granted by the Ministry of Health, Department of Research. Approval from the National Ethical Committee (012/CNE/MP/07) and from the Ethical Committee of MSF was obtained. Informed written consent was asked from all ulcer patients who participated in the survey.

## Results

Out of a total population of 103,000 inhabitants, 26,679 lived in the sampled chèferies. A total of 105 BU cases were identified. The age of the cases ranged from 2 to 75 years. The median age for active cases was 15.5 years (Interquartile range (IQR) 11–34 years) and 16 years for patients with sequelae (IQR 13–25). The female/male ratiobwas 0.8 (46/59) for all cases. Of the 105 cases identified, 49 (46.7%) presented with a sequelae and 56 (53.3%) were active cases. A total of 93 cases (88.6%) presented with one lesion and 12 cases (11.4%) with two lesions. The location of the first lesion was predominantly on the legs, 68 cases(64.8%).

Of the 56 active cases, the major or first lesion was an ulcer in 48 cases(85.7%), an oedema in 4 cases(7.1%) and a nodule in 4 cases (7.1%). The median diameter of ulcers was of 4 cm (IQR 2–7 cm) meaning that half of the ulcer cases would have been classified as category 1 or early lesions according to the new WHO categories. The median delay since beginning of the first BU symptoms for active cases was 12 weeks (IQR 3–30).

### Prevalence

**Figure 1:**
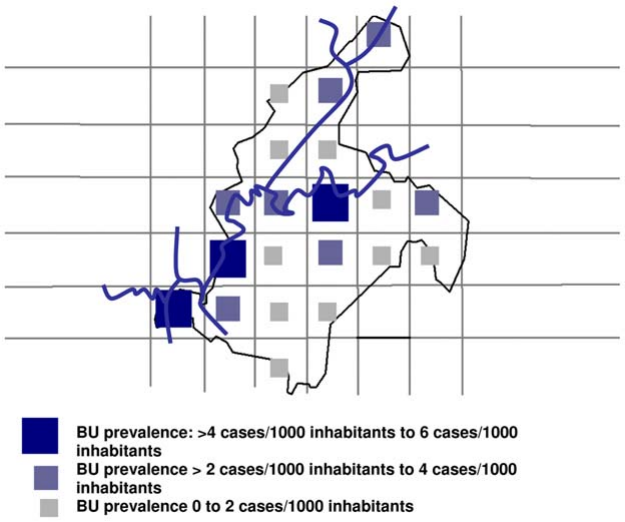
Map of Akonolinga Health district, prevalence of active BU cases, March 2007.

**Figure 2:**
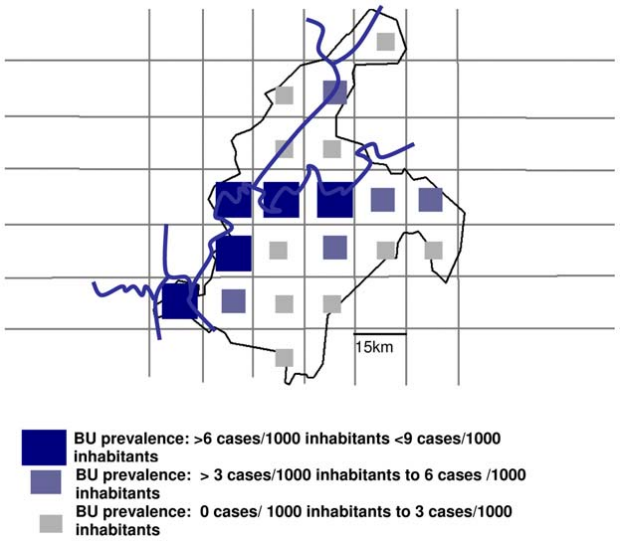
Map of Akonolinga Health district, prevalence of all BU cases, March 2007.

The overall prevalence of all BU cases was 4.7/1000 (95%CI: 4.1–7.3/1000) and the prevalence for active BU cases was estimated as 2.5/1000 (95%CI: 2.2–3.9/1000). Prevalence estimates per quadrat were categorized as low, middle or high. For active cases, categories were: 0 to 2 cases/1000; >2/1000 to 4 /1000 and >4/1000 to 6/1000 (Figure 1). For all BU cases, categories were: 0 to 3 cases/1000; >3/1000 to 6 /1000; and >6/1000 to 9/1000 inhabitants (Figure 2). The spatial distribution of prevalence estimates per quadrat showed that quadrats with high estimates were predominantly situated along the Nyong and Mfoumou rivers (Figures 1 and 2).

### Program Coverage

Of the 105 cases identified, 19 cases had visited Akonolinga Hospital resulting in an estimated coverage for the MSF BU project of 18% (95%CI 11–27%). A total of 23 patients visited the health centre at least once, leading to an estimated coverage of the peripheral MoH health centres of 22% (95%CI 15–31%). A high number of patients (77/105) consulted a traditional healer at least once, yielding a coverage of 73% (95%CI 64–81%).

Coverage estimates per quadrat were classified in 5 categories. Coverage for all BU cases for the MSF project was above 60% in the quadrat including the Akonolinga Hospital; Health centre coverage was above 60% in 3 quadrats; and above 60% in 17 quadrats for traditional healers coverage (Table 2). The same trend in coverage was seen for active BU cases. Coverage of the MSF project was 16% (95%CI 9–28%) and of the peripheral MOH Health centres was 23% (95%CI 14–36%), while coverage of traditional practitioners was 61% (95%CI 48–73%).

**Table 2:** Number of quadrats with coverage estimate for all BU cases and active BU cases, Akonolinga, Cameroon, March 2007.

Coverage estimates %	All BU cases			Active BU cases*		
	Akonolinga Hospital	Health centres	Traditional practitioners	Akonolinga Hospital	Health centres	Traditional practitioners
[0–20]	12	8	1	13	11	2
[20–40]	6	6	0	2	1	0
[40–60]	1	3	2	2	1	4
[60–80]	1	0	7	1	2	6
[80–100]	0	3	10	0	3	6

*: 2 Quadrats did not have active cases.

**Figure 3:**
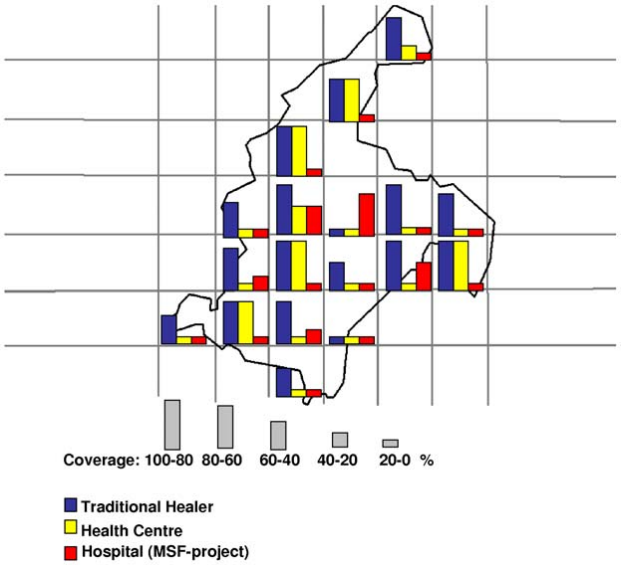
Map of Akonolinga Health district, coverage of the BU project, traditional healers and health centres, for active BU cases, March 2007.

The geographic distributions of the coverage of the three health care providers (Hospital, health centres and traditional healers) are shown in Figure 3. The quadrate including the hospital had the highest hospital coverage and the lowest traditional healer coverage.

### Sensitivity of Case Finding

We visited three chèferies (2700 inhabitants) in the health district that were not part of the survey sample to estimate the sensitivity of the case finding strategy. We found 8 cases by central location screening (M), 11 cases by combined active case finding strategy (C), and 7 cases were found by both strategies (R). The total estimated number of cases was 13. The sensitivity of the active case finding method was 84.6% (95% CI: 53.7–97.3%).

### Reasons for Not Going to the Hospital

Out of 86 patients who did not present to Akonolinga Hospital, 79 answered the question on motive for non-attendance. In the content analysis, a total of 87 reasons were coded. Twenty-five (31.6%) patients answered that they did not have enough money; 14 (17.7%) that the hospital was too far; 22 (27.8%) mentioned a lack of information; 13 were not aware of the services offered at the hospital; 9 didn't know it was free of charge; and 12 (15.2%) said that they did not want surgery.

## Discussion

Prevalence and mapping of Buruli ulcer is one of the priority areas identified by the research subgroup at the 5th WHO Advisory Group Meeting on Buruli Ulcer held in March 2002. Studies, like the one reported here, were noted as most likely to provide immediate direct benefit to Buruli ulcer patients in the medium term. The use of CSAS allowed for the identification of areas with relatively high prevalence and low coverage. An added benefit of the method is that the survey procedure also served as an awareness campaign, providing information about the disease and treatment to the population.

As previously described (4, 8), cases identified were predominantly younger than 15 years and more often male than female. This underlines once again the importance of BU as a potentially severe disabling disease that occurs at a young age. Most of the active cases (85.7%) identified presented with an ulcerative form of the disease, corresponding to what is seen at admission to the hospital (81.6%).

The overall prevalence was 0.47% (n = 105) for all BU cases including sequelae and 0.25% (n = 56) for active stages of the disease in accordance with a survey conducted previously in the region (8). Because of the lack of comparable survey in other regions it is difficult to compare our results to other estimates [18], but they corresponds to the estimated prevalence of tuberculosis in 2006 in Cameroon [19] and are slightly higher than the 6000 cases detected in a national survey in Ghana in 1999 [8]. Quadrats with higher prevalence were situated along the Nyong and Mfoumou Rivers confirming reports that cases are often found near slow moving water.

The quadrat of the area of the hospital had a high prevalence of BU, but the two other high prevalence areas were at a distance of 25 to 40 km from the hospital in the southwest of the health district.

Overall coverage of the MSF project was disappointing with 18% (12–27%) for all cases and 16% (9–18%) for active cases and was limited to quadrats neighbouring Akonolinga hospital. If we combine the geographical distribution of prevalence and coverage, we can identify the southwest of the health district as a priority area for intervention with high prevalence and low project coverage estimates. The coverage of the MOH health centres (22%) was slightly higher than the coverage of the hospital. It is important to note that this was also the case in some of the areas furthest from hospital and indicates the importance of decentralization and a functioning referral system. The high proportion of patients having visited the traditional healers, particularly in remote areas, underlines the importance of reviewing possibilities to integrate traditional healers in the project approach.

Buruli ulcer represents a financial burden for the patients and the health structures [20] Reasons given for not choosing the hospital as a health care provider were mainly financial and distance to the hospital. Lack of information about the existence of the project was also mentioned. Information on free treatment and decentralization towards health centres of diagnosis, antibiotic treatment and daily dressing, could remove barriers and rapidly improve coverage, especially when first targeting areas with high prevalence and low coverage.

A total of 15% of patients who had not gone to the hospital said it was because they did not want surgery. Providing information on the multidisciplinary combination of BU treatments (antibiotics, dressing, surgery, physiotherapy and nutrition) and giving patients the possibility to make an informed choice on which part of the treatment to accept might reduce fears and improve collaboration with traditional healers.

The quadrat including Akonolinga town presented a high prevalence and a high coverage. Since this quadrat had presumably a high population density, the overall prevalence and coverage for the health district might be slightly underestimated [11]. Population estimates of the sampled chèferies were obtained from the chiefs of the villages and confirmed by the prefecture and did not vary largely among the quadrats. Although all chiefs and all health delegates completed the same training, trust among health delegates, chiefs and villagers is a social reality that might influence the sensitivity of case finding and might vary largely. This will remain a weakness of active case finding strategies that are based on existing social networks.

In conclusion, this method was easy to use. It provided estimates of overall prevalence and coverage and identified high prevalence and low coverage areas for intervention. In addition the survey can be considered an information and awareness campaign in itself that also allowed to create a network of health delegates trained on Buruli ulcer that might refer patients in future.

## Supporting Information

Checklist S1 STROBE Checklist
(0.07 MB DOC)
